# Severity of acute hepatitis and its outcome in patients with dengue fever in a tertiary care hospital Karachi, Pakistan (South Asia)

**DOI:** 10.1186/1471-230X-10-43

**Published:** 2010-05-07

**Authors:** Om Parkash, Aysha Almas, SM Wasim Jafri, Saeed Hamid, Jaweed Akhtar, Hasnain Alishah

**Affiliations:** 1 Section of Gastroenterology, Department of Medicine Aga Khan University Hospital Karachi, Pakistan; 2 Section of Internal Medicine, Department of Medicine Aga Khan University Hospital Karachi, Pakistan

## Abstract

**Background:**

Liver injury due to dengue viral infection is not uncommon. Acute liver injury is a severe complicating factor in dengue, predisposing to life-threatening hemorrhage, Disseminated Intravascular Coagulation (DIC) and encephalopathy. Therefore we sought to determine the frequency of hepatitis in dengue infection and to compare the outcome (length of stay, in hospital mortality, complications) between patients of Dengue who have mild/moderate (ALT 23-300 IU/L) v/s severe acute hepatitis (ALT > 300 IU/L).

**Methods:**

A Cohort study of inpatients with dengue viral infection done at Aga Khan University Hospital Karachi. All patients (≥ 14 yrs age) admitted with diagnosis of Dengue Fever (DF), Dengue Hemorrhagic Fever (DHF) or Dengue Shock Syndrome (DSS) were included. Chi square test was used to compare categorical variables and fischer exact test where applicable. Survival analysis (Cox regression and log rank) for primary outcome was done. Student t test was used to compare continuous variables. A p value of less than or equal to 0.05 was taken as significant.

**Results:**

Six hundred and ninety nine patients were enrolled, including 87% (605) patients with DF and 13% (94) patients with DHF or DSS. Liver functions tests showed median ALT of 88.50 IU/L; IQR 43.25-188 IU/L, median AST of 174 IU/L; IQR 87-371.5 IU/L and median T.Bil of 0.8 mg/dl; IQR 0.6-1.3 mg/dl. Seventy one percent (496) had mild to moderate hepatitis and 15% (103) had severe hepatitis. Mean length of stay (LOS) in patients with mild/moderate hepatitis was 3.63 days v.s 4.3 days in those with severe hepatitis (P value 0.002). Overall mortality was 33.3% (n = 6) in mild/moderate hepatitis vs 66.7% (n = 12) in severe hepatitis group (p value < 0.001). Cox regression analysis also showed significantly higher mortality in severe hepatitis group (H.R (4.91; 95% CI 1.74-13.87 and P value 0.003) and in DHF/DSS (5.43; CI 1.86-15.84 and P value 0.002). There was a significant difference for the complications like Bleeding (P value < 0.001), Acute Renal failure (ARF) (P value 0.002), Acalculus cholecystitis (P value 0.04) and encephalopathy (P value 0.02) in mild/moderate and Severe hepatitis groups respectively.

**Conclusion:**

Severe hepatitis (SGPT>300IU) in Dengue is associated with prolonged LOS, mortality, bleeding and RF.

## Background

Dengue fever is an arboviral infection transmitted by Aedes ageptyi as well as Aedes Albopictus and causes 4 spectra of illness which are an asymptomatic phase, acute febrile illness, classic Dengue fever (DF), Dengue Hemorrhagic Fever (DHF) which includes Dengue Shock Syndrome (DSS) [1-3]. Dengue viral infection has been recognized as one of the world's biggest emerging epidemic. Throughout the tropics this infection has an annual incidence of 100 million cases of DF with another 250,000 cases of DHF and mortality rate of 24,000-25,000 per year[1,4,5]. Most of these cases are reported from South East Asian areas which are most favorite tourist's point[4,6]. In Pakistan at least two confirmed outbreaks have been reported first in 1994 and second in 2005. Since then we are continuously facing the problem of an epidemic each year in Karachi and some other areas of Pakistan [7,8]. During the last couple of years Pakistan has become an endemic region as this virus is being transmitted westward from India[7].

Dengue fever indicates a bad outcome including death when liver and nervous system are involved simultaneously by dengue[9]. Atypical manifestations include liver involvement, central nervous involvement (encephalopathy) and cardiac alterations in DF. None of the studies have reported about the severity of liver involvement in Dengue fever with reference to mortality and Length of Stay (LOS). Liver involvement in dengue fever is manifested by the elevation of transaminases representing reactive hepatitis. This has been recognized over the last two decades during the recent epidemics in Brazil[10]. A study from Thialand (Retrospective review) has reported 34.6% liver dysfunction in pediatric population infected with dengue[11]. Elevated transaminases in DF are due to many conditions like use of hepatotoxic drugs and direct attack of virus itself causing these unusual clinical manifestations. These in turn lead to more serious fate among the dengue patients [10,12].

The involvement of liver in dengue fever is not uncommon as reported in literature since 1970[13]. In the Liver Function Tests (LFT) most common abnormality seen is elevated transaminases which are involved in amino acid metabolism. In approximately 90% of the patients with DF, Aspartate Aminotransferase (AST) is higher than the Alanine Aminotransferase (ALT) [14,15]. DF initiates the inflammatory responses leading to liver parenchymal changes and causing release of transaminases in circulation[16]. Since DF is an emerging infection in Pakistan and very little is known about the severity of hepatitis and their outcome in patients with dengue fever when liver is involved. Therefore we aim to assess the frequency of hepatitis in dengue infection. We also aim to compare their outcome (mortality, length of stay and complications) between patients with mild to moderate and severe hepatitis in dengue infection.

## Methods

We conducted a cohort study on inpatients with dengue fever at Aga Khan University Hospital Karachi Pakistan (AKUH). All patients were uniformly tested on day of admission using a standardized protocols being used in hospital for patients with dengue infection. We selected a cohort of patients with hepatitis from these patients with dengue infection. AKUH is part of Aga Khan Development Network aimed at providing high quality of health care, teaching and research. This University Hospital has 542 beds in operation and provides services to over 38,000 hospitalized patients and to over 500,000 outpatients annually with the help of professional staff and facilities that are among the best in the region.

Ethical clearance was taken from the institutional Ethical Review Committee (ERC). All inpatients of age ≥ 14 years who had history of acute fever, positive dengue IgM and whose ALT were done, were included in the study. We had excluded all those patients who had underlying Chronic Liver Disease (CLD) or known positive serology for viral hepatitis (HBsAg or HCVAb; n = 6) and those patients who had malaria.

We divided this cohort of dengue inpatients with hepatitis into two groups based on ALT level; Mild (upto 5 times normal) to Moderate (5- 10 times) acute hepatitis: ALT 23-300 IU/L, severe acute hepatitis: ALT > 300 IU/L or ≥ 10 times [10,17-21]. Primary outcome measures were mortality and LOS. Secondary outcome measures were different complications; defined as; a) Bleeding was defined as either mucosal bleeding (epistaxis or gum bleed) or Gastrointestinal (GI) bleed; b). Acute Renal Failure (ARF) was defined as rise of creatinine (Cr) >3 times of normal [22]; c) Encephalopathy was defined as altered mental status (drowsiness, lethargy, agitation, or coma) for ≥ 8 hours [23]; d) Shock was defined as systolic pressure < 90 mm Hg [24]; e) Acalculous cholecystitis was defined as inflammation of gall bladder without stone on ultrasound[2]. We also dichotomized the data based on diagnosis into DF and DHF/DSS for the primary outcome measures (mortality and LOS) [25].

We required a minimum sample size of 350 patients by assuming 35% prevalence of liver dysfunction in patients with dengue fever, with 0.05 bound on error(precision) and 95% confidence level[11].

Data was collected on predesigned proforma which included demographics, clinical presentation, laboratory parameters and outcome (in hospital mortality, length of stay and complications). We included all patients who fulfilled inclusion criteria and were admitted in the medical ward through emergency room or clinic. Informed consent was taken from patients. Proforma was filled for each patient. Each patient was categorized either into mild to moderate or severe hepatitis groups based on ALT levels and these patients were followed for their outcome till the end of inpatient stay.

### Statistical analysis

Statistical Package for Social Sciences (SPSS) version 16 was used for analysis. Results are presented as mean ± standard deviation (SD) for continuous variables, frequency and percentage are given for qualitative variables. For non normally distributed quantitative variables median and Inter Quartile Ranges (IQR) were used. Chi square test was used to compare categorical variables and fischer exact test were used when numbers were too small to perform the chi-square testing. Student t test was used to compare continuous variables. In survival analysis cox proportion hazard ratio (Cox regression) was determined for mortality in DHF/DSS and severe hepatitis after fulfilling the assumption of proportional hazard ratio. Kaplan Meir plot of survival over time and log rank test were also used. A p value of ≤ 0.05 was taken as significant. Death rates for 10 days in each category were calculated and 95% CI for death rate ratio were also calculated.

## Results

Total of 699 patients with DF were included, among them 65% were male. Mean age of patients was 31.87 ± 13.55 years. Approximately 86% (n = 604) were admitted with DF, 12% were admitted with DHF and only 2% had DSS. Malarial parasite was negative in all patients. Mean duration of fever was 6 ± 3.27 days with mean temperature of 38.5 ± 1°C. Among clinical features, 48.6% had nausea/vomiting, 18.2% had abdominal pain, 17% had rash, 16% had body ache, 10% had diarrhea, 9% had cough, 5.4% had hematemesis, 5.2% had bleeding gum,4.3% had malena,4% had altered mental status and 3.1% had jaundice. On abdominal examination, 7% had right hypochondric tenderness and 1.4% had epigastric tenderness. In hemodynamics, mean pulse was 96 ± 18.5 beats/min, mean systolic blood pressure was 113 ± 16 mmHg with mean diastolic blood pressure of 71 ± 11 mmHg. The complete blood count showed mean hemoglobin of 13.71 ± 2.37 gm/l, mean hematocrit of 40.07 ± 6.8%, mean white blood cell count of 5.5 ± 5/cmm and median platelet count of 48/cmm;IQR 23-96/cmm. Mean Activated Partial Thromboplastin Time (APTT) was 36.34 ± 11.56 seconds with control of 40 seconds. Mean Cr was 1.12 ± 1 mg/dl. Liver function tests (LFTs) show the median ALT of 88.50 IU/L; IQR 43.25-188 IU/L, median AST of 174 IU/L; IQR 87-371.5 IU/L, median ALK.Phos 80 IU/L; IQR 54-129 IU/L and median T.Bil of 0.8 mg/dl; IQR 0.6-1.3 mg/dl. Almost 86% (599) of the patients had elevated ALT (hepatitis). The mean LOS was 3.88 ± 2.7 days in this population. Mortality was 2.7% in our study. Among complications, we have found 1.7% bleeding, 1.3% shock, 2.7% ARF 3.9% acalculous cholecystitis and 2.9% encephalopathy.

**Figure 1:**
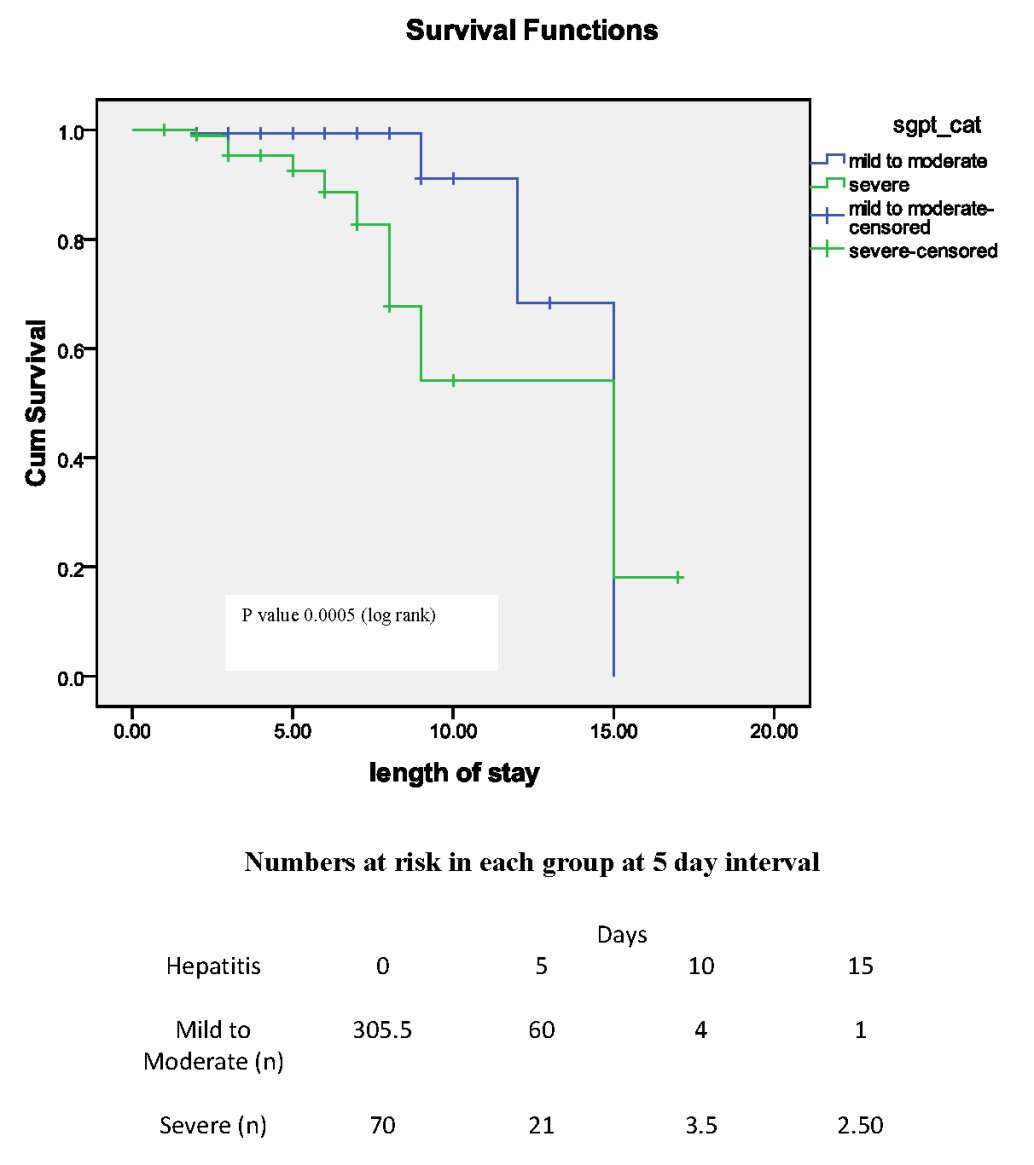
**Kaplan-Meir curve of survival over time in two groups of patients with hepatitis**.

**Figure 2:**
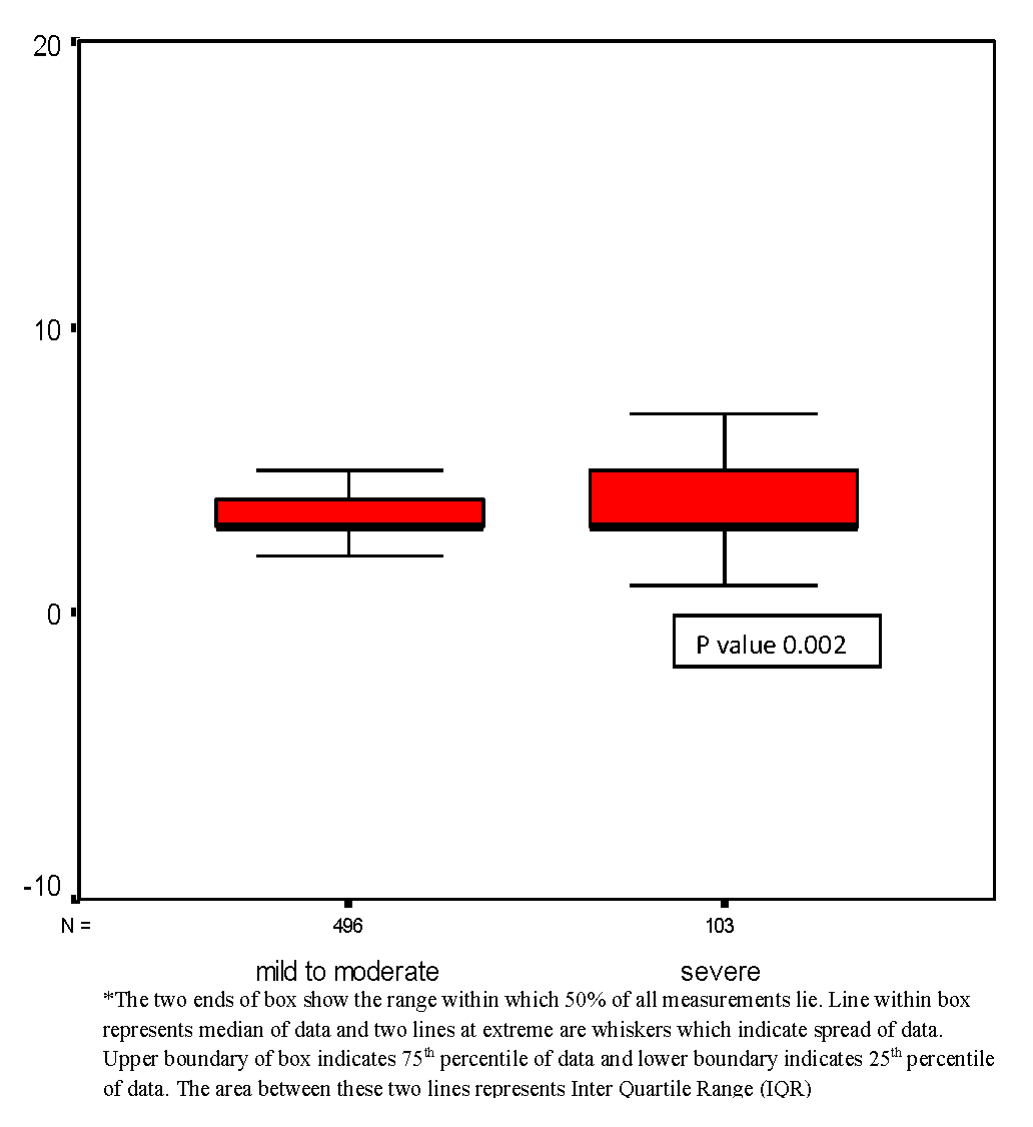
**Box plot showing length of stay (in days) in two groups of severity of hepatitis**.

Severe hepatitis was present in 15% and mild to moderate hepatitis in 71% while 14% had normal ALT. Patients with normal ALT were excluded from further analysis. Overall mortality (n = 18) was significantly higher in severe hepatitis group (66.70%;n = 12) as compared to mild to moderate hepatitis (33.30%;n = 6). The survival probability of mild to moderate and severe hepatitis group according to Kaplan-Meir curve is shown figure 1 with numbers at risk below the figure. Differences in overall survival between these two groups is significant (P value < 0.005; log rank). Mean length of stay in patients with mild/moderate hepatitis was 3.63 days versus 4.3 days in those with severe hepatitis (figure 2). Cox regression (survival analysis) also showed the significantly higher mortality in severe hepatitis group (H.R 4.91; 95% CI 1.74-13.87 and P value 0.003) and DHF/DSS group (H.R 5.43; CI 1.86-15.84 and P value 0.002) with reference to mild to moderate hepatitis and DF respectively. Subgroup analysis of DF and DHF/DSS for mortality has shown significantly higher mortality in severe hepatitis group versus mild to moderate hepatitis (3/436 versus 3/81, P value 0.04 in DF; 3/60 versus 9/22, p value < 0.001 in DHF/DSS group respectively). Comparison of subgroups DF and DHF/DSS with reference to outcome is shown in table 1. Comparison of secondary outcome measures is shown in table 2.

**Table 1:** Primary outcome measures according to severity of hepatitis within two categories diagnosis

Diagnosis Categories	Severity of hepatitis	Deaths n	Death rate (Deaths/10 day)	H.R (95% CI; UCI, LCI)^	P value (Difference in death rate)!	LOS ± SD	P value
Dengue fever (n = 517)	Mild to Moderate Hepatitis (n = 436)	3	0.019	2.97(0.48,18.6)	0.283	3.50 ± 1.54	<0.025
	Severe hepatitis (n = 81)	3	0.093			3.96 ± 2.27	
DHF/DSS (n = 82)	Mild to Moderate Hepatitis (n = 60)	3	0.010	5(1.3,16)	0.003	4.60 ± 2.87	.212
	Severe hepatitis (n = 22)	9	0.073			5.60 ± 3.85	

^H.R = Hazard Ratio, UCI = Upper confidence interval, LCI = Lower confidence interval

! Log Rank value

**Table 2:** Complications in two groups of severity of hepatitis

Complications	Mild to moderate (n = 496)	Severe hepatitis (n = 103)	P value
**Bleeding^1 ^(n)**	3	7	<0.001^6^
**Renal failure^2 ^(n)**	8	8	0.002
**Encephalopathy^3 ^(n)**	7	11	0.02
**Shock^4 ^(n)**	6	2	0.40^6^
**Acalculus cholecystitis^5 ^(n)**	14	7	0.04

1. Either mucosal bleeding (epistaxis or gum bleed) or Gastrointestinal (GI) bleed

2. Acute Renal Failure (ARF) was defined as rise of creatinie >3 times(13)

3. Encephalopathy was defined as altered mental status for > 8 hours (drowsiness, lethargy, agitation, or coma)(19)

4. Shock was defined as Hypotension (defined as systolic pressure < 90 mm Hg)(14)

5. Acalculus cholecystitis was defined as inflammation of gall bladder without stone on US (15)

6. Anayzed by fischer exact test

## Discussion

We report the largest cohort study from south Asia (Pakistan) on hepatitis in dengue fever patients and their association with outcome. Currently dengue is perhaps the most common rapidly emerging viral infection throughout the world and Pakistan reported its first outbreak in 1994[26].

Deranged liver functions are common in patients with dengue infection due to direct attack on liver cells or unregulated host immune response against the virus [19]. Hence measurement of AST and ALT are mandatory to see the liver involvement [10]. We have found in our study that deranged liver functions are an important feature in patients with dengue infection. Almost 86% of the patients in our study had hepatitis (elevated ALT) and 95% patients had elevated AST. However Wong et al reported that AST abnormality was predominantly higher as compared to ALT; 91% and 72% respectively, which is consistent with our study. However we report higher values of AST and ALT in our study population than reported by Wong et al and other studies [10,19,27,28]. Souza et al also reported the similar trend of AST/ALT in dengue fever but with much lower level as compared to our population[10]. This difference can be explained on the basis that in our study all patients were inpatients while in their study all the patients were outpatients with less severe disease. Furthermore a study from Asia pacific region (Taiwan) by Kuo et al has shown approximately 90% of the AST abnormality in dengue patients which is consistent with our study[29]. We can assume that reasons for higher ALT or AST levels in our population are either due to more virulent strain of dengue infection or virus is more hepatotoxic. Therefore further studies are required to highlight the possible hepatotropic nature of this virus as well as virulence and type of virus.

Immune responses including the innate and acquired; play important role in determining the response to any virus. Causation of the severity dengue infection has been explained by the hypothesis of immune enhancement and virulence nature of virus [21]. Chaturvedi et al in their study detected the appearance of different helper cells cytokines in human white blood cells cultures infected in vitro with dengue virus type 2. In their study they have reported that during dengue infection; monocytes, B cells, T cells and mast cells produce large amounts of cytokines [30]. Based on this we can hypothesize the similar mechanism responsible for hepatoxicity in our study population. Despite all this, role of host immunity in dengue infection is still very unclear. Dengue antigens have been identified within the liver parenchyma on postmortem of these patients hence virus seems to be able to replicate within the hepatocytes. Unregulated host immune response may play some part in severity of dengue infection therefore by modifying the immune response, severe infection can be prevented [21].

We have seen significantly higher mortality in patients with severe hepatitis. We report 2.7% mortality in our study population. Similar mortality figure (2.6%)was reported in an earlier study from Pakistan but with a smaller sample size (n = 225)[31]. Literature regarding the mortality in dengue infection with impaired liver function is scarce. Nguyen TL et al concluded in their study on 45 patients in Vietnam, that DHF may cause mild to moderate liver dysfunction in most cases; only some patients may suffer from acute liver failure leading to encephalopathy and death [9]. However liver involvement was not studied with reference to mortality. Shah has reported high mortality in dengue patients with hepatitis and encephalopathy[32]. However this was a small study from India, done on 4 pediatric patients only. Our study is the first large study from South Asia region, to highlight the mortality with deranged liver function in dengue patients. The self limiting clinical course of dengue infection can be prolonged when the liver is involved as suggested by Souza et al[10]. Hence we have seen worse prognosis in terms of mortality and length of stay in our study patients when there is a severe hepatic involvement. On further subgroup analysis we also saw that mortality was significantly higher in severe category of hepatitis in both DF and DSS. We can postulate that severe hepatitis may be a significant contributing factor to mortality in patients with dengue infection. However further immunologic studies need to be done to establish the cause of liver involvement; whether it is due to virus itself or due to immunologic reaction (reactive hepatitis).

The mean length of stay was approximately 4 days in our study population with dengue infection while a study from Singapore by Lye et al[33] had reported mean LOS of 3 days. Khan et al reported in their study (166 patients) in Saudi population; that median duration of hospital stay was 4 days in dengue patients but there was no comparison based on liver functions [34]. No study has reported LOS in comparison with liver involvement as yet. Severe hepatitis in dengue fever does affect the LOS when the disease is mild (DF) but as disease become more severe, severe hepatitis does not affect the LOS.

We have found that clinical complications like bleeding, renal failure, encephalopathy and Acalculous cholecystitis were higher in those who had severe hepatitis. A study from Saudi population by Khan et al had made an association between high AST level with these complication[34]. But we have seen the complications with reference to hepatitis based on serum ALT levels. We have seen significantly higher bleeding complication in patients with severe hepatitis. Similarly Kuo CH et al has reported higher bleeding episodes in those who had high levels of AST, ALT and GGT[29]. An other study has also reported significantly higher spontaneous bleeding episodes in patients with elevated ALT and Alk.Phos [27]. Nguyen TL et al has also found significantly higher elevation of AST and ALT in DHF patients with gastrointestinal haemorrhage [9]. We can postulate that deranged liver functions may have a significant role in bleeding in addition to thrombocytopenia. We have found significantly higher proportion of patients with renal failure who had severe hepatitis. There are case reports of acute renal failure/injury in dengue fever in the literature, but none of them have been studied along with liver involvement in adults[35]. Encephalopathy in our study population was also significantly higher in the severe hepatitis group. There are case reports available regarding the encephalopathy in dengue infection in adult population[36]. However encephalopathy is well studied in pediatric population[37]. Acalculous cholecystitis was significantly high in the patients with severe hepatitis. Acalculous cholecystitis is very known entity among patients with dengue fever but none of them have shown comparison according to severity of hepatitis[38,39].

Three percent of the patients had jaundice in our study population. Larreal Y et al has reported jaundice in only 2 out of 63 patients in their study on hepatic alteration in dengue fever [40].

## Strength and Limitations

This is the largest study from South Asia on patients with dengue fever. This is the first study which is comparing the outcome in dengue patients with hepatitis.

This study has limited external validity since this is study of inpatients and has not included patients who have visited outpatient and other hospitals in the city. The study population in our tertiary care hospital is from middle to high income population which comprises only small percentage of city population. We also did not check for the type of dengue virus.

## Conclusion

We conclude that majority of the patients with dengue infection have hepatitis. Severe hepatitis in dengue infection has got worse outcome in terms of length of stay, mortality and complications as compared to mild to moderate hepatitis. Therefore severe hepatitis can be considered as a bad prognostic indicator of outcome in dengue infection. Though majority of the patients had self resolving course of illness but they can be potential candidate for acute fulminant hepatic failure. Dengue fever should be considered when liver functions are deranged apart from routine hepatotropic viruses. Further studies are required to establish the fact whether liver injury is due to hepatotropic nature of the virus or due to immunologic injury.

## New to knowledge

Outcome of dengue fever when there is an involvement of abnormal liver functions.

## List of abbreviations

ALT: (Alanine aminotransferase); AST: (Aspartate aminotransferase); GGT: (Gamma glutamine transferase); Alk.Phos: (alkaline phosphate); IU: (International units); DHF: (Dengue hemorrhagic fever); DF: (Dengue fever).

## Competing interests

The authors declare that they have no competing interests.

## Authors' contributions

OP has made the protocol and overall plan of the study. OP has written the final manuscript. AA had reviewed the manuscript first draft and helped in revision of article based on reviewers comments each time. AA also helped in coordinating this study. HAS has been the great supervisor who reviewed the protocol and had given important suggestions during study as well as in revisions. WJ has given the moral support. All other authors have reviewed and helped in drafting the manuscript.

## Authors Information

1. Dr Om Parkash (OP); MBBS, FCPS (Medicine)

Resident Gastroenterology, Section of gastroenterology Department of Medicine Student of Masters' in clinical research Aga Khan University Hospital Karachi.

2. Dr Aysha Almas (AA); MBBS, FCPS (Medicine), MSc in Clinical Research

Senior Instructor, Section of internal Medicine Department of Medicine

3. Professor S.M Wasim Jafri (WJ); MBBS, FRCP, FRCPE

Gastroenterologist and hepatologist, Department of Medicine

4. Professor Saeed Hamid; MBBS, FRCP

Gastroenterologist and hepatologist, Department of Medicine

5. Professor Javeed Akhtar; MBBS, FRCP

Professor of Medicine and section head of Internal Medicine.

6. Professor Syed Hasnain Alishah (HAS); MBBS, FRCP

Gastroenterologist and Hepatologist

Head Section Gastroenterology, Department of Medicine

## Pre-publication history

The pre-publication history for this paper can be accessed here:

http://www.biomedcentral.com/1471-230X/10/43/prepub
